# The Treatment of Sleep Dysfunction in Neurodegenerative Disorders

**DOI:** 10.1007/s13311-020-00959-7

**Published:** 2020-11-11

**Authors:** Zanna J. Voysey, Roger A. Barker, Alpar S. Lazar

**Affiliations:** 1 Department of Clinical Neurosciences, John van Geest Centre for Brain Repair, University of Cambridge, Cambridge, CB2 0PY UK; 2 Department of Clinical Neurosciences, John van Geest Centre for Brain Repair and WT-MRC Cambridge Stem Cell Institute, University of Cambridge, Cambridge, CB2 0PY UK; 3 Faculty of Medicine and Health Sciences, University of East Anglia, Norwich, NR4 7TJ UK

**Keywords:** Sleep, Insomnia, Neurodegeneration, Dementia, Alzheimer’s, Parkinson’s

## Abstract

Sleep dysfunction is highly prevalent across the spectrum of neurodegenerative conditions and is a key determinant of quality of life for both patients and their families. Mounting recent evidence also suggests that such dysfunction exacerbates cognitive and affective clinical features of neurodegeneration, as well as disease progression through acceleration of pathogenic processes. Effective assessment and treatment of sleep dysfunction in neurodegeneration is therefore of paramount importance; yet robust therapeutic guidelines are lacking, owing in part to a historical paucity of effective treatments and trials. Here, we review the common sleep abnormalities evident in neurodegenerative disease states and evaluate the latest evidence for traditional and emerging interventions, both pharmacological and nonpharmacological. Interventions considered include conservative measures, targeted treatments of specific clinical sleep pathologies, established sedating and alerting agents, melatonin, and orexin antagonists, as well as bright light therapy, behavioral measures, and slow-wave sleep augmentation techniques. We conclude by providing a suggested framework for treatment based on contemporary evidence and highlight areas that may emerge as major therapeutic advances in the near future.

## Introduction

Sleep dysfunction is prominent across the spectrum of neurodegenerative conditions, occurring in 40 to 90% of patients [1–5]. It is a key determinant of patients’ quality of life [6], falls risk [7], and progression to institutionalization [8–11], as well as caregiver burden and health [12–14]. It should therefore be a priority in the clinical care of patients with neurodegenerative disease but is currently typically overlooked.

Moreover, mounting recent evidence contends that sleep dysfunction contributes to the severity and progression of neurodegeneration [15]. On a symptomatic level, it is well-established that sleep deprivation and disruption leads to deficits in attention, executive function, and processing speed, as well as promoting impulsivity, emotional lability, and depression [16–20]. Sleep abnormalities therefore almost certainly contribute to the burden of such features common to neurodegenerative conditions. Importantly, however, exacerbation may also occur at the level of the underlying pathogenic processes. The discovery of the glymphatic pathway and its predominant activity during sleep [21–23], for example, has revealed the critical role of sleep in the clearance of extracellular neurotoxic waste linked to some forms of neurodegeneration. Clearance of both β-amyloid and tau has been shown to occur in this way [24, 25], and sleep deprivation has been shown to effect marked, rapid increases in these pathogenic protein species in both animal models [26, 27] and humans [28–30]. Likewise, sleep deprivation has been shown to promote tau propagation [27] and induce neuroinflammation [31], both of which have been implicated in neurodegenerative pathophysiology. Other recent studies have also revealed in detail the role of sleep in memory consolidation through synaptic modelling and the formation of cortical engrams [32–34]. Impairment of sleep is thus very likely to contribute to the synaptic dysfunction and memory impairment characterizing many neurodegenerative conditions.

Addressing sleep dysfunction in neurodegeneration is therefore of critical importance, offering potential to not only enhance quality of life for patients and their families, but also to mitigate disease burden and delay progression. Given the lack of therapies that currently achieve this for neurodegenerative conditions, this potential is of paramount significance.

Here, we review the latest evidence for traditional and emerging sleep therapies in neurodegeneration, both pharmacological and nonpharmacological. Reflecting the weight of available literature, considered evidence relates predominantly to Alzheimer’s and Parkinson’s disease, but, where available, is also discussed in relation to Huntington’s disease, Lewy body dementia, and vascular dementia.

## Sleep Dysfunction in Neurodegenerative Conditions: Commonalities and Discriminators

Important commonalities are present among neurodegenerative conditions with regard to the nature of sleep dysfunction. Insomnia, in the form of delayed sleep onset, fragmentation, or early awakening difficulties, is a prevalent abnormality affecting around 50% of patients [4] and is convergent across the spectrum of neurodegenerative disease [4, 35, 36]. Circadian dysfunction is also common throughout, as evidenced by loss of robust rest–activity rhythms, abnormalities of melatonin concentration and rhythmicity, and loss of autonomic diurnal fluctuation [37, 38]. Likewise, where sleep architecture is analyzed via polysomnography, a characteristic pattern of abnormalities emerges; that of increased arousals/awakenings, loss of slow-wave and rapid eye movement (REM) sleep stages, increased latency to initiation of REM, and consequent gain in time spent in the lightest stages of sleep [35, 39–41]. It is also notable that, across the spectrum of neurodegenerative disease, sleep abnormalities frequently precede diagnostic clinical features: for example, sleep fragmentation precedes the onset of overt Alzheimer’s disease and manifest Huntington’s disease [42, 43] and REM behavior sleep disorder (RBD) often precedes the clinical onset of motor features of Parkinson’s, multiple system atrophy, or Lewy body dementia [44, 45].

Nevertheless, important discriminators are also evident. For example, obstructive sleep apnea (OSA) is notably prevalent in Alzheimer’s disease, affecting 40% of patients [46, 47], and is the predominant abnormality seen in vascular dementia [48]. By contrast, restless leg syndrome (RLS) and RBD are more prevalent in Parkinson’s disease, affecting 15 and 35 to 46% patients respectively [2, 5, 49], with rates of RBD in multiple system atrophy and Lewy body dementia being even higher [35]. While daytime sleepiness and sundowning are prominent in Alzheimer’s disease, true excessive daytime sleepiness and sleep attacks typify Parkinson’s disease [5, 50]. Periodic limb movement disorder, meanwhile, is seen more commonly in Huntington’s disease [51].

These parallels and discrepancies likely reflect the underlying pathology of neurodegeneration. Commonalities are likely driven by shared neurodegenerative patterns of hypothalamic involvement leading to circadian dysfunction, and generalized atrophy leading to an inability to gate and support sleep stages appropriately. By contrast, discriminators likely reflect relative patterns of disease burden, for example, a brainstem emphasis in Parkinson’s disease, *versus* basal forebrain dysfunction in Alzheimer’s [52].

## Preliminary Therapeutic Considerations

It is vital that the process of addressing sleep dysfunction in a patient with neurodegeneration begins with a comprehensive assessment of the specific problems in question. For an excellent recent review of appropriate questions, validated scales, and objective investigations such as sleep studies and actigraphy to aid in this process, please see the recent review by Ooms and Ju [48]. This process will help ensure identification of specific clinical sleep pathologies where present and recognition of preliminary contributory factors to sleep dysfunction such as depression, pain, nocturia, medications, and caffeine/nicotine/alcohol use. It is imperative that such elements are addressed first, prior to consideration of other strategies.

### Depression

Depression is highly prevalent in neurodegeneration [53], and while the relationship between depression and sleep dysfunction is bidirectional, it is well recognized that depression frequently leads to initiation and maintenance insomnia, as well as sleep architectural disturbance including a loss of slow-wave sleep [54]. Treating depression in Parkinson’s disease has been associated with improvements in sleep symptoms [55, 56]. Therefore, where depression and sleep dysfunction are concomitant and the severity of depression mandates treatment, it is prudent to consider such treatment prior to other sleep interventions. However, attention should be paid to the selection of antidepressant: selective serotonin reuptake inhibitors and venlafaxine can impair sleep quality, whereas agents such as trazodone and mirtazapine promote sleep [54], and antidepressants can exacerbate clinical sleep pathology (Table 1).

**Table 1:** Key considerations in addressing clinical sleep pathology common in neurodegenerative conditions

Pathology	Example of associated neurodegenerative condition	Key treatment strategies
Obstructive sleep apnea (OSA)	1. Alzheimer’s disease 2. Vascular dementia	1. Continuous positive airway pressure (CPAP) 2. Mandibular devices 3. Positioning techniques
Restless leg syndrome (RLS)	1. Parkinson’s disease	1. Exclude iron/B_12_ deficiency 2. Consider contribution of antihistamines/antidepressants/ antipsychotics 3. Dopamine agonists, gabapentin, pregabalin 4. Opiates
Periodic limb movement disorder (PLMD)	1. Huntington’s disease [51]	1. Consider contribution of antidepressants/antipsychotics 2. Dopamine agonists
REM behavior sleep disorder (RBD)	1. Parkinson’s disease 2. Lewy body dementia 3. Multiple system atrophy	1. Consider contribution of antidepressants, beta blockers, opioids, clonidine 2. Melatonin [57–59] 3. Clonazepam (except in presence of cognitive impairment/OSA)

### Pain

Contributory pain, for example due to comorbid osteoarthritis, should be addressed with analgesics. Discomfort due to rigidity/akinesia, dystonia, and impaired bed mobility in Parkinson’s disease are commonly thought to contribute to sleep dysfunction [60], such that dopaminergic therapy may be of benefit [61]. However, some studies effecting specific improvement in these features have failed to show concomitant improvement in sleep quality [62, 63].

### Nocturia

Nocturia is a prominent cause of sleep disruption in Parkinson’s disease, affecting around 35 to 66% of individuals [5, 49], but is also a contributory factor in many patients with other neurodegenerative conditions often secondary to age-related comorbid conditions such as benign prostatic hypertrophy or heart failure. Simple measures such as the optimization of timing of diuretics, reduction of water intake from late afternoon, or convene catheter systems can therefore be of significant benefit to many patients with disrupted sleep. In the setting of Parkinson’s, optimization of dopaminergic therapy may effect improvement in nocturia [64, 65], particularly the nocturnal use of rotigotine patches [66]. Anticholinergic interventions for nocturia, however, should be viewed with caution due to their well-documented deleterious cognitive side effects.

### Influence of Medications for Comorbidities

The contribution of medications for comorbid conditions should also be considered. For example, α-blockers, β-blockers, corticosteroids, serotonin selective reuptake inhibitors, and angiotensin-converting enzyme inhibitors all have detrimental effects on sleep and should therefore be rationalized in the setting of sleep dysfunction.

### Influence of Medications for Neurodegenerative Conditions

The contribution of medications administered for the neurodegenerative condition itself should also be considered, especially with respect to timing and dose. For example, donepezil and memantine administered for Alzheimer’s have been shown to improve sleep quality overall [67, 68] but donepezil may cause sleep disruption if taken late in the day [69]; modification of timing may therefore bring benefit. Likewise, dopamine agonists improve sleep quality and daytime functioning in Parkinson’s [70, 71] but carry a dose-related risk of excessive daytime somnolence and sleep attacks; therefore, dosing should be reviewed in the setting of sleep dysfunction.

### Caffeine, Nicotine, and Alcohol Exposure

Exposure to caffeine, alcohol, or nicotine less than 4 h prior to going to bed is associated with poor sleep quality; patients’ use of such items should therefore also be reviewed and addressed. Particular effort should be made to probe alcohol use accurately, as 26% of elderly individuals with sleep problems have reported self-medicating with alcohol for sleep induction [72], and alcohol abuse is frequent in neurodegenerative conditions that promote addictive behaviors/impulse control disorders.

### Treatment of Clinical Sleep Pathology

Alongside such considerations, clinical sleep pathology such as OSA, RLS, PLMD, or RBD should be addressed in a targeted way where present. This is vital, as such factors make a significant contribution to overall sleep dysfunction: for example, RLS frequently leads to sleep onset insomnia, and OSA and PLMD may contribute to sleep fragmentation and daytime sleepiness [73, 74]. This is also important as highly effective interventions are available: for example, continuous positive airway pressure (CPAP) for OSA has been shown not only to improve sleep quality, but also to enhance mood and cognition, and slow cognitive decline in Alzheimer’s disease [75–78]. The treatment of such pathology has been recently reviewed in detail elsewhere [49], but a framework of key strategies is provided in Table 1.

Where such interventions are unsuccessful in effecting sufficient improvement in sleep dysfunction, further pharmacological and nonpharmacological strategies should be considered. Herein we evaluate current evidence in these two domains.

## Pharmacological Therapies

### Sedating and Alerting Agents

A number of hypnotic agents, including benzodiazepines, Z-drugs, and sedating antidepressants and antihistamines, are frequently used to treat insomnia in healthy adults. However, such agents are almost exclusively only approved for short-term use and are recommended as a secondary measure to nonpharmacological approaches. This is because i) all such agents bear limited efficacy and salient risk of significant adverse effects and ii) there is insufficient evidence to balance benefits against such risks for long-term use in healthy adults [79]. There is even less scope to contend that benefits outweigh such risks in older individuals or patients with neurodegenerative disorders: as outlined below, robust randomized controlled trials are sparse, and extrapolation from observational studies suggests that risks are likely to be more prominent in such populations.

#### Benzodiazepines and Z-Drugs

Both benzodiazepines, such as temazepam, nitrazepam, and triazolam, and Z-drugs, such as zopiclone, zolpidem, and zaleplon, improve sleep quality by reducing sleep latency and awakenings via agonism of GABA_A_ receptors, the main inhibitory neurotransmitter receptors in the brain. Adverse effects of benzodiazepines include tolerance, dependence, and withdrawal, as well as both acute [80, 81] and chronic [82] impairment of attention and psychomotor speed. Impairment of memory consolidation is also well supported, although refuted by some studies [83–85]. While risks of such adverse effects are lower with Z-drugs, they are not absent [86, 87]. Moreover, both benzodiazepines and Z-drugs have been found to reduce REM and/or slow-wave sleep [88–90], stages of sleep vital to memory consolidation [32–34] and glymphatic clearance of neurotoxic waste [21].

In line with these observations, among elderly populations, benzodiazepines and Z-drugs are consistently associated with an increased risk of hip fracture due to falls [91], and some evidence suggests an increased risk of cognitive impairment [92] and decline [93]. Likewise, in an observational study in a probable Alzheimer’s cohort adjusted for baseline cognitive function and confounding clinical features [94], use of benzodiazepines was associated with a higher risk of deterioration. In Parkinson’s disease, a small placebo-controlled study of eszopiclone identified improved subjective sleep measures without marked adverse events [55], but with a treatment period of only 6 weeks. High-quality evidence among neurodegeneration-specific cohorts is otherwise lacking.

Thus, it is highly likely that use of such agents in a neurodegenerative population will bring harm in excess of benefit and should therefore be viewed with caution.

#### Sedating Antihistamines and Antidepressants

Sedating antihistamines such as diphenhydramine and hydroxyzine are some of the most frequently used agents in individuals with insomnia, in part due to their availability in over-the-counter preparations. However, the mixed antihistamine/anticholinergic effect of such agents can cause prominent sedation, cognitive impairment, delirium, and increased daytime somnolence, such that avoidance is recommended in a neurodegenerative population [95–97].

Sedating antidepressants, such as amitryptiline, trazodone, mirtazapine, and doxepin, are also commonly used in the treatment of insomnia. Of note, trazodone has shown neuroprotective effects and rescue of memory deficits in preclinical models of neurodegeneration [98]. However, the majority of such antidepressants bear the same potential for deleterious outcomes as antihistamines, due to mixed anticholinergic and/or antihistamine effects. In line with this, observational studies in older individuals have identified the use of such agents as associated with increased risk of both hip fracture [99] and mortality [100]. A small blinded placebo-controlled trial of trazodone in an Alzheimer’s disease cohort found an improvement in total sleep time without prominent adverse events or cognitive impairment [101]. However, the treatment period was only 2 weeks, this finding has not been replicated in other studies, and, indeed, a number of other studies have identified trazodone as bearing prominent risks of cognitive impairment [102] and adverse outcomes [100]. Alongside this, a randomized controlled trial of mirtazapine in Alzheimer’s has found no benefit to sleep or cognitive outcomes, with an increase in daytime drowsiness [103]. It is therefore recommended that such agents are avoided for sleep therapy except where depression is concomitant and requires pharmacological intervention. Nevertheless, some more favorable evidence is present for doxepin: a 12-week double-blind controlled study in elderly individuals with chronic primary insomnia identified objective sleep benefit without prominent adverse effects [104], and an open-label study in a Parkinson’s cohort identified improved cognitive and subjective sleep outcomes without major adverse effect [105], although the treatment period was only 6 weeks. Again, however, these findings have yet to be replicated or trialled in larger cohorts, and as such this evidence has to be seen to be as inconclusive at this stage.

#### Antipsychotics

Antipsychotics represent a further class of agents that have been used in the treatment of sleep dysfunction in neurodegeneration, typically capitalizing upon their hypnotic effect when administered for behavioral and psychological symptoms of dementia (BPSD). Short-term randomized studies have supported their ability to significantly increase total sleep time in patients with dementia [106], and a 5-year observational study in Alzheimer’s disease comparing outcomes from risperidone *versus* zolpidem, melatonin, or no treatment found superior outcomes within the risperidone subgroup [107]. However, this is contradicted by the appropriately adjusted observational study conducted by Ellul et al. [94], in which antipsychotics were associated with greater deterioration in patients with Alzheimer’s, and is at odds with a weight of evidence suggesting that antipsychotics carry an increased risk of hip fracture [99], cognitive decline, cerebrovascular accident, and death [99, 108–110] among patients with dementia. It is therefore not recommended that such agents are used to address sleep dysfunction, unless they already mandated for concomitant BPSD. Where this is needed, there is some evidence to suggest that quetiapine carries a lower risk of adverse effects than other antipsychotics, but this is tempered by concurrent lower efficacy for treating BPSD [111].

#### Sodium Oxybate

Sodium oxybate, an agent used in the treatment of narcolepsy and thought to act via GABAergic mechanisms, is attracting growing interest as a possible alternative pharmacological agent for the treatment of sleep dysfunction in neurodegeneration. Not only has it been found to enhance slow-wave activity and counteract the cognitive effects of sleep deprivation in healthy adults [112], it has also shown promising effects on toxic proteinopathies, neuroprotection, and neuroinflammation in animal models of neurodegeneration [113]. Moreover, in Parkinson’s disease, both open-label [114] and small randomized controlled trials [115] have identified both subjective and objective sleep improvement following its use. Nonetheless, *de novo* sleep apnea or parasomnia was induced in 3/12 participants [115], long-term use in a neurodegenerative cohort has yet to be assessed, and its proposed GABAergic mode of action raises the potential that this agent will have similar problems to those seen with benzodiazepines and Z-drugs.

#### Modafinil

Some studies have considered the converse approach to addressing sleep dysfunction in neurodegeneration, by administering the alerting agent modafinil during waking hours. However, evidence to date has not supported its use: modafinil has failed to show consistent benefit for fatigue [116] or daytime somnolence [114, 117, 118] in Parkinson’s disease or for apathy in Alzheimer’s disease [119]. A small randomized controlled acute single-dose trial in Huntington’s disease identified some improvement in alertness, but with no improvement to mood, and deleterious cognitive effects [120].

### Melatonin

Melatonin, the hormone that helps orchestrate circadian sleep–wake function, has received considerable research interest as a further alternative pharmacological agent for the treatment of sleep dysfunction in neurodegeneration. Exogenous melatonin administration can influence both circadian rhythm and sleep quality [121], and the reduction in release of endogenous melatonin across the spectrum of neurodegeneration [37] would suggest that administration may have a beneficial normalizing effect. Moreover, preclinical studies have suggested that melatonin could have an independent antioxidant, anti-inflammatory, and neuroprotective role [122], and one of promoting glymphatic clearance [123] of neurotoxic waste.

However, while a number of studies have suggested a benefit of melatonin therapy in Alzheimer’s disease, the great majority of these are open-label studies and based on subjective outcomes [124–127]. Of the five robust randomized controlled trials in Alzheimer’s disease employing objective outcomes based on actigraphic sleep variables, the evidence is inconclusive. Three have failed to show benefit to any sleep variable [128–130], while two have demonstrated mixed benefit: Asayama et al. [131] reported a significant increase in total sleep time (mean, 103 min) with decreased nocturnal activity, while Dowling at al. [132] reported improved rest–activity rhythm, but only through improvements in wake activity rather than sleep, and only when administered in conjunction with light therapy. This lack of consensus may relate to the wide range of melatonin doses employed in these studies. The cognitive benefit of melatonin therapy in Alzheimer’s disease has been reported in two studies [131, 133] but the extent of this is of questionable clinical significance: a change of 1.5 out of a total of 30 points on the Mini-Mental State Examination (MMSE) and 3.5 to 4.3 out of a scale of 70 points on the Alzheimer’s Disease Cognitive Assessment Scale (ADAS-Cog).

Only one robust study of melatonin therapy has been undertaken in mild cognitive impairment. This double-blind placebo cross-over study [134] suggested improvement in both sleep quality and rest–activity rhythms as reported by actigraphy outcomes, as well as benefits to depression and cognition, but to our knowledge this finding has not been reproduced in any other study to date.

Very limited high-quality data is available as to the value of melatonin therapy in Parkinson’s disease. Of studies available in English, there has been one placebo-controlled trial with actigraphic outcomes [135] which demonstrated no benefit other than a gain of just 10 min of total sleep time, while a randomized double-blind placebo-controlled trial showed no benefit on objective polysomnographic outcomes and no benefit to motor symptoms [136]. However, a recent evidence-based review [137] has found melatonin to be “possibly useful” in the treatment of insomnia in Parkinson’s, on the basis of i) positive studies employing subjective sleep outcomes and ii) melatonin’s low side effect profile.

Overall, meta-analyses evaluating the effect of melatonin on Alzheimer’s, Parkinson’s, or mixed dementia have found no benefit to any sleep or cognitive variable [138, 139], other than two reporting a modest, likely clinically insignificant increase in total sleep time: 24 min [140] and 33 min [141].

However, these findings should not be conflated with those pertaining to RBD, where there is a strong evidence base for the efficacy of melatonin: open-label prospective [57, 59], retrospective [58], and double-blind placebo-controlled [142] studies have all suggested significant scope for melatonin therapy to reduce RBD episodes, as confirmed by polysomnographic outcomes.

### Orexin Antagonists

Orexin, also known as hypocretin, is a key hypothalamic neuropeptide regulating sleep by promoting wakefulness and arousal. The recent development of pharmacological orexin antagonists for clinical use therefore represents an emerging avenue of sleep therapy bearing potential in neurodegeneration [52, 143]. In addition, preclinical studies in mice have suggested that orexin antagonism can also suppress β-amyloid accumulation [144].

Two phase 3 trials of orexin antagonists [145, 146] have produced moderately promising results in the treatment of primary insomnia, increasing total sleep time (55 min treatment group *vs* 19 min placebo group) and reducing wake time after sleep onset (48 min treatment group *vs* 25 min placebo) by reducing the duration of wakeful periods. Moreover, head to head trials of orexin antagonists *versus* zolpidem have suggested superiority with respect to latency to persistent sleep and sleep maintenance [147] and also to cognitive outcomes [148]. Levels of somnolence appear comparable [149] and, if present, are mild–moderate and rarely require drug withdrawal [145, 146]. Sleep architecture also appears largely unaffected [145].

Encouragingly, studies involving older healthy participants have not demonstrated significantly different efficacy or safety outcomes [145, 150], and the sole trial in a neurodegenerative cohort that has been published to date has suggested strikingly similar outcomes to those in healthy cohorts. In this randomized controlled trial undertaken in patients with insomnia and mild–moderate Alzheimer’s [151], orexin antagonism was superior to placebo with respect to improvements in total sleep time (73 min *vs* 45 min) and wake time after sleep onset (− 45 min *vs* – 29 min), with no major effect on sleep architecture. Moreover, limited cognitive assessment through the Mini Mental State Examination and Symbol Digit Task did not suggest deleterious cognitive outcomes, somnolence was not prominent or severe, and while there were more falls in the treatment group (2.1% *vs* 0%), these were not judged to be drug related.

Nonetheless, the efficacy and safety of orexin antagonists in neurodegeneration remains to be tested robustly. Indeed, the fact that orexin levels are reduced in many neurodegenerative conditions [152, 153] may predict poor outcomes, and the central activity of orexin antagonists raises the potential for adverse events in such populations. In particular, the phase 3 studies were undertaken in cohorts that excluded depression. Given the high rates of coincidence of depression and sleep dysfunction in neurodegenerative conditions, assumptions cannot yet be extrapolated regarding safety for affective outcomes. It is also of note that, to date, orexin antagonists are only approved for clinical use in Japan, Australia, and the USA.

## Nonpharmacological Interventions

Nonpharmacological sleep therapies, most notably bright light therapy and behavioral interventions, also merit consideration.

### Bright Light Therapy

Light is the most powerful zeitgeber of the human circadian system [154], and bright light therapy delivered by light emitters (e.g., lightboxes) is known to affect both circadian rhythm [155, 156] and sleep quality [157] in healthy individuals. There is also a strong evidence base for its efficacy in depression [158]. The potential for bright light therapy to treat sleep dysfunction and its corollaries in neurodegeneration has therefore received considerable research attention.

The majority of studies with objective actigraphic sleep outcomes have been undertaken in mixed dementia cohorts in residential settings. Most have demonstrated a degree of benefit; many in the absence of a control arm raising the possibility of placebo effects [159–161], but gains have also extended to controlled studies [162–164]. The most common benefit has been of strengthened circadian rhythm [159, 160, 164, 165], but some studies have also demonstrated enhanced total sleep time and sleep efficiency [159, 161] and reduced wake after sleep onset [161]. Five studies have also analyzed cognitive outcomes [166–170]. Each demonstrated minor benefit, but outcomes were limited to Mini-Mental State Examination (MMSE) only and may have been consequent to the alerting influence of bright light rather than via changes in sleep quality. Evidence regarding the effect on behavioral features of neurodegeneration is mixed: while some studies have reported improvements in both depression and agitation alongside sleep benefit [159, 166], others have demonstrated exacerbated agitation [171].

With respect to studies in specific dementia subtypes, studies of bright light therapy in Alzheimer’s-only cohorts have demonstrated similar benefits to those in mixed dementia studies [163], while a head-to-head study of vascular dementia *versus* Alzheimer’s disease favored a greater benefit in vascular dementia [162]. Uncontrolled, unblinded studies in Parkinson’s disease have suggested benefit of bright light therapy to mood, motor outcomes, and subjective markers of sleep [172, 173]. However, controlled studies have found frequent parallel sleep outcomes between active and control conditions [174, 175], raising the possibility of a placebo effect.

Despite this overall positive potential, meta-analyses of bright light therapy in neurodegeneration have largely failed to recommend its use overall. While the benefits seen in most studies are acknowledged, these have been insufficient to contend significant efficacy overall [176–178]. One meta-analysis has advocated its use [179], but estimates a weak effect size (Hedges *g* = 0.3). This outcome likely relates to the same factors that limit the practical suitability of bright light therapy for patients with neurodegenerative conditions: to be both effective and comparable between studies, bright light therapy requires consistent daily use, at a consistent time tailored to the patient’s circadian rhythm, at a consistent sedentary distance to achieve uniform lux exposure, and in a season/setting where effects are not outweighed by environmental light exposure [166]. This is clearly not always feasible for patients with dementia; indeed, studies incorporating adherence analysis have reported noncompliance to be as high as 25% (155; Lazar personal communication). The development of blue light glasses [180] may circumvent some of these barriers, but have yet to be tested robustly.

### Behavioral Measures

Behavioral measures, collectively known as cognitive behavioral therapy for insomnia (CBT-I) and summarized in Table 2, have a strong evidence base in improving sleep quality in healthy individuals [181].

**Table 2:** Behavioral sleep therapy measures

Measure	Description
Stimulus control	Avoidance of bed other than for sleeping or sex; leaving bed if unable to sleep
Sleep restriction	Limiting the sleep period to promote consolidated sleep
Sleep hygiene	Regular bed/wake and meal times, limited daytime napping, optimization of bedroom temperature/light/noise, avoidance of nicotine/caffeine/alcohol < 4 h before bed, regular physical exercise > 4 h before bedtime
Cognitive therapy	Altering deleterious psychological response to poor sleep
Relaxation techniques	Including meditation, guided imagery, and progressive muscular relaxation

Studies of the use of such measures in neurodegeneration have been encouraging. As for bright light therapy, the majority of evidence comes from mixed dementia cohorts, most using multicomponent techniques focusing on sleep hygiene. Almost all studies have suggested benefit, many in enhanced daytime wakefulness [182, 183], but also in reduced wake time at night [184] and sleep duration [185], confirmed both by actigraphy [182–185] and polysomnography [186, 187]. Some studies have also reported associated cognitive benefit [186] and a reduction in agitation [185, 188].

Moreover, such findings extend to Alzheimer’s specific cohorts, where both sleep hygiene alone [189] and in combination with light therapy [190, 191] have been shown to benefit sleep quality. Similarly, CBT-I has been shown to benefit sleep quality and executive function in mild cognitive impairment [192]. Akin to bright light therapy, there is a relative paucity of high-quality studies in Parkinson’s specific cohorts, with most studies employing subjective outcomes suggesting benefit [193–195], whereas objective measures have tended to be negative [105, 196]. Nonetheless, daytime physical exercise has been found to improve nocturnal actigraphy [197] and polysomnographic outcomes [198] in Parkinson’s.

As with bright light therapy, the application of such measures is clearly contingent upon an individual patient and/or carer’s ability to implement/engage with them consistently, which may be impractical for many patients. Adherence is key to efficacy [191], and attrition rates have been as high as 40% in community studies [199]. Furthermore, in the main, carers have been found to need tailored and patient-specific advice rather than generic guidance [200], which may limit feasibility.

A combined recent meta-analysis [176] of bright light therapy and behavioral measures in the treatment of sleep dysfunction in neurodegeneration has reported benefit to sleep efficiency, but only when used in combination. However, no overall benefit was found for cognition, agitation, depression, or functional outcomes.

## Slow-Wave Sleep Augmentation

Slow-wave sleep (SWS) represents the central electrophysiological element of the sleep-dependent recovery process, as well as being implicated in sleep-dependent memory consolidation [32], age-related changes in cognition [201], and glymphatic clearance of neurotoxic waste [23, 202]. It is consistently impaired across the spectrum of neurodegeneration [35]. As a result, the development of neurophysiological stimulation techniques that augment SWS, most notably transcranial direct-current stimulation (TDCS) and acoustic stimulation applied during sleep, is a further important emerging frontier in the field of sleep therapies in neurodegeneration.

The ability of such techniques to augment the slow oscillations (SOs) underpinning SWS has been demonstrated consistently and robustly in numerous blinded cross-over sham/stim paradigm studies [203]. While it may seem improbable that pulses of sound alone would have such an effect, it is possible and thought to be mediated by an interaction between the nonlemniscal acoustic pathway and diffuse thalamocortical projections during sleep, wherein acoustic stimuli evoke a widespread synchronous response, akin to a transient SO, in order to mediate arousal to threat. Where the stimulus is at threshold, it is able to induce SO activity without arousal [204].

### Transcranial Direct-Current Stimulation

Augmentation of slow oscillation activity by TDCS has demonstrated benefit to declarative memory in both young [205–207] and older [208, 209] healthy participants, and in both nocturnal [206] and nap [205, 207, 208] protocols. In Cellini et al. [207], this was accompanied by an overall increase in time spent in slow-wave sleep, and cognitive benefits persisted at 48 h.

There have, however, been a number of negative studies using this approach [210–212]. This discrepancy may relate to the variability of the stimulation protocols applied, as well as the timing and frequency of TDCS—all of which appears to be critical to efficacy. For example, Marshall et al. [213] found that theta-TDCS reduces slow oscillation activity and impairs declarative memory while the stimulation protocol used in the negative study by Eggert et al. [212] in fact induced a reduction in slow-wave sleep and increased wake time. By contrast, Cellini et al. [207] found that stimulation during asynchronous slow-wave sleep was more efficacious than that applied during endogenous SO activity, and positive findings may have related to the incorporation of such stimulation in their study. Incongruent findings may also relate to the study population: Koo et al. [214], for example, found that any cognitive benefits of TDCS were only apparent when participants were stratified according to higher baseline memory function.

Of note, two studies also evaluated the effect of sleep–TDCS on mood, demonstrating a positive effect in both healthy participants [205] and those with schizophrenia [215].

To date, only one study of TDCS in sleep has been conducted in a neurodegenerative cohort. This study [216], undertaken during a nap protocol in sixteen participants with mild cognitive impairment, demonstrated benefit to visual but not verbal or visuospatial declarative memory at 30 min post awakening.

### Acoustic Stimulation

Slow-wave augmentation by acoustic stimulation has likewise demonstrated cognitive benefit in young [217–219], middle-aged [220], and older healthy adults [221], in both nap [217] and nocturnal [218–221] protocols, with benefits seen in executive function [220] as well as declarative memory [217–219, 221]. In most cases, this was mediated through tones of pink noise delivered by headphones, but more recently, bone conduction via a mastoid application has also been implemented successfully [222].

Compellingly, the degree of cognitive enhancement correlated with the degree of slow-wave augmentation elicited in a number of these studies [218, 220, 221]. Response rates appear variable, however, and in some studies, benefit was only seen when nonresponders were excluded (35% participants) [220]. As with TDCS, there are studies failing to demonstrate cognitive benefit within the literature, but these have typically also failed to show an overall increase in slow-wave activity following the intervention [223, 224]. The precise stimulation protocol again appears critical, but in comparison with TDCS, efficacy to boost slow-wave activity and benefit to cognition appears greatest when stimulation is phase-locked to the upstate of endogenous SOs [218, 225, 226]: so-called closed loop stimulation.

There has to date been only one study of acoustic augmentation of SWS in a neurodegenerative cohort [227]. Conducted in 2019, this nocturnal pilot study in nine patients with amnestic mild cognitive impairment demonstrated highly variable increases in slow-wave activity (1-30%), but promisingly, improvement in declarative memory was demonstrated in five of nine participants, and the degree of improvement correlated with that of slow-wave activity augmentation.

The ease of delivery in a domiciliary setting, and its low side effect profile, places acoustic stimulation at a significant practical advantage over TDCS. Notably, a number of companies have now developed headband devices delivering closed loop acoustic stimulation, designed for use during sleep in the home setting (e.g., Dreem2 headband, Phillips SmartSleep Deep Sleep Headband), in some cases combined with delivery of personalized behavioral measures guidance. It is worth noting, however, that the cumulative effects of the long-term application of acoustic stimulation on cognition are not known: all studies evaluating cognitive outcomes have, as yet, incorporated single-night and immediate postsleep testing protocols only.

## Concluding Remarks

Sleep dysfunction in neurodegenerative conditions merits prioritization in clinical care: not only because it has the potential to significantly improve quality of life for patients and their families, but also because there is mounting evidence to suggest that it may also mitigate cognitive and affective symptoms and slow disease progression. Given the lack of therapies that currently achieve this in neurodegeneration, this potential should not be discounted.

In all cases, it is important that the approach to treating sleep dysfunction in a patient with a neurodegenerative condition begins with a comprehensive assessment of this aspect of their condition, via targeted questions, objective scales, and investigations, in order to identify preliminary contributory factors (depression, pain, nocturia, medications, and caffeine/nicotine/alcohol use) and specific clinical sleep pathology (OSA, RLS, RBD, PLMD) where present. These should be addressed with conservative measures and specific treatments as outlined.

Only in the event of such measures failing to bring adequate benefit should other interventions be considered. Of available secondary measures, current evidence is most in favor of behavioral measures alongside bright light therapy, although it is clearly imperative that the practicality of such measures for an individual’s circumstance is taken into account. Currently, the landscape of pharmacological options is largely unfavorable, with deleterious effects likely to outweigh benefit for sedating agents including antidepressants, antihistamines, and antipsychotics, and a lack of cogent evidence suggesting significant benefit from modafinil or melatonin. We would advocate that sedating agents are only used where already mandated for concomitant affective or behavioral symptoms, or in the event of failure of all other measures. The overriding message is therefore that, as clinicians, we should resist the engrained temptation to treat sleep dysfunction in neurodegeneration by add-on prescription and instead take time to implement conservative measures and nonpharmacological interventions that are more likely to bring benefit. Nonetheless, the landscape of pharmacological options is evolving, with both sodium oxybate and orexin antagonists showing promise. Finally, the development of domiciliary devices providing slow-wave sleep augmentation provides further hope of efficacious intervention emerging in the near future.

### Required Author Forms

Disclosure forms provided by the authors are available with the online version of this article.
